# New Mathematical Model for the Surface Area of the Left Ventricle by the Truncated Prolate Spheroid

**DOI:** 10.1155/2017/6981515

**Published:** 2017-05-04

**Authors:** José Sérgio Domingues, Marcos de Paula Vale, Carlos Barreira Martinez

**Affiliations:** ^1^Department of Mechanical Engineering, Federal University of Minas Gerais (UFMG), Belo Horizonte, MG, Brazil; ^2^Department of Mathematics, Federal Institute of Minas Gerais (IFMG), Formiga, MG, Brazil; ^3^Cardiovascular Surgery, Nossa Senhora das Dores Hospital, Itabira, MG, Brazil

## Abstract

The main aim of this study was the formula application of the superficial area of a truncated prolate spheroid (TPS) in Cartesian coordinates in obtaining a cardiac parameter that is not so much discussed in literature, related to the left ventricle (LV) surface area of the human heart, by age and sex. First we obtain a formula for the area of a TPS. Then a simple mathematical model of association of the axes measures of a TPS with the axes of the LV is built. Finally real values of the average dimensions of the humans LV are used to measure surface areas approximations of this heart chamber. As a result, the average superficial area of LV for normal patients is obtained and it is observed that the percentage differences of areas between men and women and their consecutive age groups are constant. A strong linear correlation between the obtained areas and the ventricular volumes normalized by the body areas was observed. The obtained results indicate that the superficial area of the LV, besides enabling a greater knowledge of the geometrical characteristics of the human LV, may be used as one of the normality cardiac verification criteria and be useful for medical and biological applications.

## 1. Introduction

The human heart possesses four chambers: right atrium, left atrium, right ventricle, and left ventricle (LV). The right atrium receives deoxygenated blood and pumps it to the right ventricle, which compresses itself, pumping the blood toward the lungs, where it is oxygenated and, thus, it returns to the heart, coming in by the left atrium, and then it is moved to the LV, which, on its turn, compresses itself, forcing the blood to flow to all the body's arteries, in order to oxygenate the cells and withdraw their impurity [1].

Due to its big influence on the general functioning of the heart, many diagnoses of diseases are based on determinations of cardiac parameters related to the LV. Because of its high capacity of presenting adaptive answer to a series of physiopathological processes and cardiovascular diseases, such as chronic renal failure, hypertension, and heart disease, the LV has become the focus of many researches for the improvement of the existing methods and obtainment of new methods of normality verification and heart disease diagnoses [2, 3].

Some of the main cardiac parameters for the LV, which allow verifying the normality of the heart function, are as follows: ventricular volume, ejection fraction, shortening fraction, and ventricular mass. Generally, they are based on the fact that this chamber possesses a similar geometry to the ellipsoid of revolution, or, still, to a truncated prolate spheroid (TPS), implying that two of its axle shafts must have equal measures [4–9].

Once the blood volume ejected by the LV and its structural composition is essential to the good functioning of the heart, it is natural that the parameters related to volume and mass, as already mentioned, are the focus of scientific researches.

The surface area of the LV and other heart chambers is a cardiac parameter rarely discussed in the literature. Feng et al. [10] presented an interesting discussion upon the determination of this parameter, using real data of 10 patients and nuclear methods of image obtainment. However, the patient's data are not available, not even characteristics as sex, age group, and body area. The mathematical model that was used considers that the LV has the shape of a TPS and is based on ellipsoidal coordinates, leading the surface area calculation to depend on three spatial variables. Theses variables are obtained through a relatively simple manner, by nuclear methods, but they are of complex obtainment when one wants to use, for instance, echocardiographic images. In the search for a way which allows this calculation with a simpler obtainment of the model variables, we used the Cartesian coordinates for the TPS in this work.

In Krajcik and McLenitham [11], (1) is presented for calculation of the surface area of an arbitrary ellipsoid by the Cartesian coordinates, and it depends on the incomplete elliptic integrals of the first and second orders, *F*(*φ*, *k*) and *E*(*φ*, *k*), respectively, and of the axle shafts *a* > *b* > *c* > 0.

(1) Sa,b,c=2πc2+2πba2−c2a2−c2Eφ,k+c2Fφ,k,

(2) φ=sin−1a2−c2a2,

Equation (1) is a readaptation of the result described in Bowman [12], which is also described in the work of Lawden [13]. In Krajcik and McLenitham [11] and Tee [14] equivalent readaptations of (1) are presented as well, verified in 1714 by Roger Cotes, for the situation where *c* > *a* = *b* = *r* > 0, as it is seen in (3), which is a particular case of ellipsoid of revolution, denominated as prolate spheroid, presenting the result of sin^−1^ given in radians.

(3) $$\begin{array}{c} S\left(\;c,r,r\;\right)=2\pi r^{2}+\frac{2\pi rc^{2}}{\sqrt{c^{2}-r^{2}}}\sin^{-1}\sqrt{\frac{c^{2}-r^{2}}{c^{2}}}. \end{array}$$

Recently, advancement in the ellipsoid theory was observed, in which the surface area of the general ellipsoid with its three axles with different values was calculated in an analytically closed way in terms of Appell's generalized hypergeometric functions [15]. However, since the normal LV has its two short axles with practically identical measurements and only one apex, it is believed that the geometry of a general ellipsoid is no longer adequate for this association, and it can lead to the overestimation of the values obtained for its internal surface area. Besides, experimental analysis shows better result accuracy when the predefined geometry for the LV is of a truncated ellipsoid [8, 9].

So, in this work, we could start the discussion on the possible values ranges for the surface area of the LV in people considered normal (without diagnosed heart problems), which is the main focus of this research, and also, to compare the results with the ones described in [10], we deduce an equation to calculate, precisely, the surface area of a TPS, once we had no reliable references for a formula of this kind of surface. Furthermore, we developed a mathematical model that associates real data of the LV dimensions of normal patients with the dimensions of a TPS and, then, we have obtained approximations of the surface area of this cardiac chamber, so important for the functioning of the circulatory system.

## 2. Material and Methods

### 2.1. The Surface Area of the Truncated Prolate Spheroid

**Figure 1:**
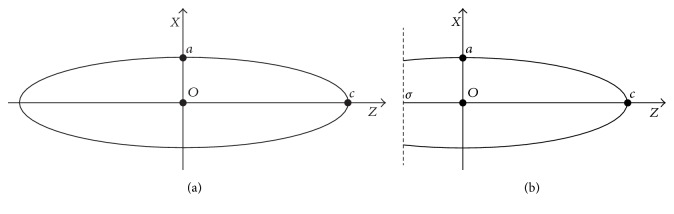
Ellipse centered in the origin and axle shafts *a* and *c*, in (a). In (b), truncated ellipse by the line *z* = *σ*.

Consider the ellipse in plane *ZX*, centered in the origin (Figure 1(a)), with bigger and smaller axle shafts, *c* and *a*, respectively, defined by

(4) $$\begin{array}{c} \frac{x^{2}}{a^{2}}+\frac{z^{2}}{c^{2}}=1. \end{array}$$

Take also the line *z* = *σ*, where *σ* ∈ *ℝ*. It defines a cut in this curve, generating a truncated ellipse, as Figure 1(b). Thus, it should be (*z*, *x*) ∈ Ω = [*σ*, *c*] × [−*a*, *a*].

From (4) it is possible to write the variable *x* on function of *z*, which represents the part of the truncated ellipse, where *x*(*z*) ≥ 0.

(5) $$\begin{array}{c} x\left(\;z\;\right)=a\sqrt{1-\frac{z^{2}}{c^{2}}}. \end{array}$$

Thus, when performing the revolution of the curve given by (5) around axis *Z*, a TPS is obtained, once its axle shafts will be *a*, *b*, and *c*, with *c* > *a* = *b* and *σ* ≤ *z* ≤ *c*.

**Figure 2:**
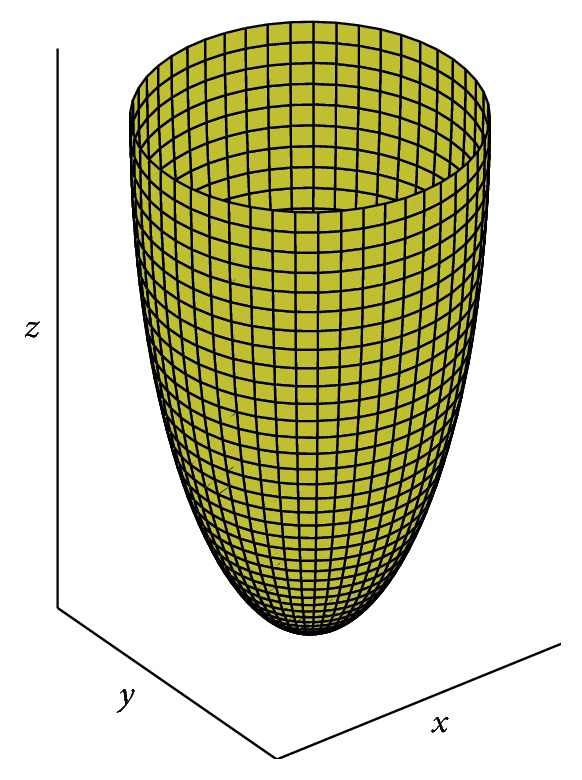
TPS generated by the rotation if the curve is defined by (5).

*A*_*σ*_(*S*) area of the surface described in Figure 2 can be calculated in this way:

(6) $$\begin{array}{c} A_{\sigma }\left(\;S\;\right)=2\pi \overset{c}{\underset{\sigma }{\int }}x\left(\;z\;\right)\cdot \sqrt{1+{\left[x^{\prime }\left(z\right)\right]}^{2}}dz. \end{array}$$

From (6) we have

(7) AσS=2πa∫σc1+a2−c2z2c4dz=2πac22c2−a2sin−1⁡zc2−a2c2+z2c2c4+a2−c2z2σc=2πac22c2−a2sin−1⁡cc2−a2c2+c2c2c4+a2−c2c2−c22c2−a2sin−1⁡σc2−a2c2−σ2c2c4+a2−c2σ2=πac2c2−a2sin−1⁡c2−a2c−c2c2−a2sin−1⁡σc2−a2c2+1cc4+a2−c2c2−σc2c4+a2−c2σ2=πac2sin−1⁡c2−a2c−sin−1⁡σc2−a2c2c2−a2+1cc4+a2c2−c4−σc2c4+a2−c2σ2

(8) ∴AσS=πac2sin−1⁡c2−a2c−sin−1⁡σc2−a2c2c2−a2+a−σ1+a2−c2σ2c4,

which is the formula for the surface area of a TPS.

Notice that (8) is just in function of the plane coordinates, *a*, *c*, and *σ*, obtained by the intersections of the TPS with its coordinated axes.

It is clear that a prolate spheroid can be considered as the union of two TPSs and that is the idea we will use to present an example that shows the validity of (8), based on the result given by (3).

**Example 1 .**

**Figure 3:**
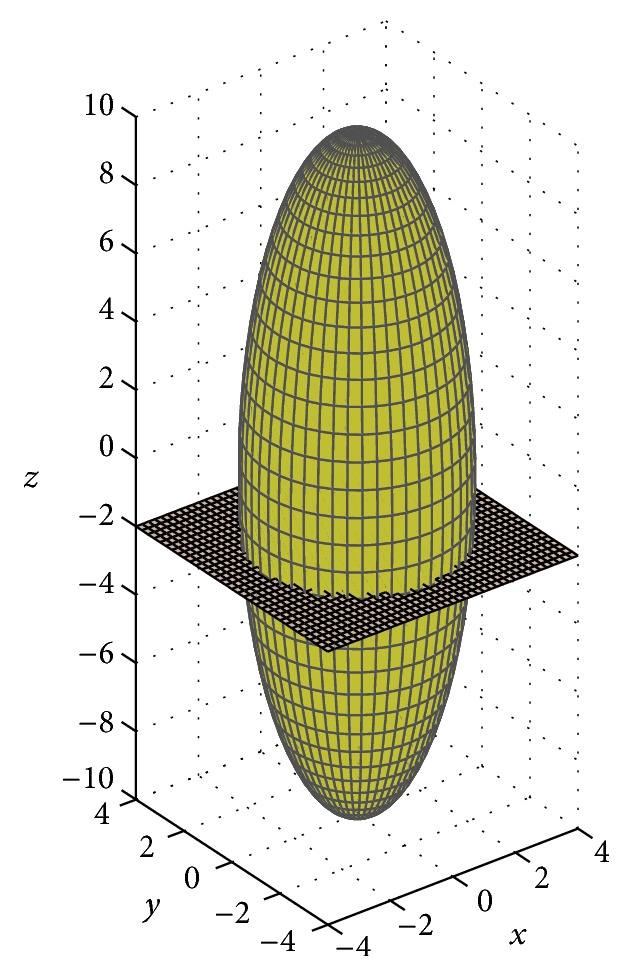
Representation of plane *z* = −2 splitting the prolate spheroid into two TPSs, to demonstrate the validity of (8), shown in this work.

Consider a prolate spheroid of axle shafts *c* = 10 cm and *a* = *b* = 3 cm. By using (3), the surface area of this figure is *S*(10, 3, 3) = 306.73 cm^2^. Taking the particular case, in which *σ* = −2, and with the same axle shafts, this prolate spheroid can be considered as the junction of two TPSs, obtained through the cut generated by plane *z* = −2, as highlighted in Figure 3.

By using the symmetry property of the prolate spheroid, we have that the surface area of the TPS on the left in Figure 3 is equal to the area of the TPS in which *σ* = 2. Therefore, when we use (8), the sum of the surface areas of the regions on the left and on the right, generated by plane *z* = −2, is

(9) $$\begin{array}{c} A_{2}\left(\;S\;\right)+A_{-2}\left(\;S\;\right)=190.83+115.90=306.73\;{cm}^{2}, \end{array}$$

which matches the value of *S*(10, 3, 3) and shows the validity of (8).

The next subsection is dedicated to the association of the necessary measures to determine the values of the surface area from (8) with the LV axes measures.

### 2.2. Mathematical Model for the Association of the Measures *a*, *b*, *c*, and *σ* to the LV Axes

**Figure 4:**
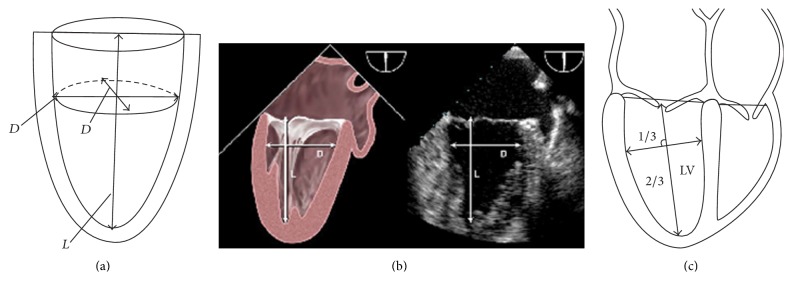
We have, in (a), an adaptation of a figure withdrawal from [8]. In (b), with an adaptation from [16], on the left, we have a more realistic picture of the LV and, on the right, there is a real image obtained through echocardiography. In both cases, the adequate way of obtaining the measures is highlighted. In (c), adapted from [17], one of the possibilities of obtaining the measures is presented, in which it is established that *D* value must be obtained at the point that divides *L* into 1/3 and 2/3 proportions.

Two important cardiac parameters of the LV are as follows: the measure of the* long axis *(*L*) and the* short axis *(*D*), Figure 4, with the latter being immediately taken at or below the ends of mitral valve leaflets [16, 19, 20]. The developed model considers that the LV has the geometry of a TPS and, for this reason, it will present a way of writing *a*, *b*, *c*, and *σ* measures of the TPS from measure *D*, which is easily obtained in echocardiographic or nuclear tests.

In Figure 4(a), it is observed that both diameters that represent the LV short axis are equal to *D*, once it is being considered as a TPS [8]. Besides that, while observing Figure 4(b), *L* can be considered as the length of the real interval [*σ*, *c*], which allows writing that

(10) a=b=D2,

(11) c+σ=L.

One can also consider that the intersection of the segments represented by *L* and *D* determines the origin of plane *ZX*. Therefore, the largest portion generated in *L* division will represent the measure of the largest *c* semiaxis, and the other portion will represent |*σ*| measure. Of course, as *L* > *c*, whenever *σ* < 0, according to what is described in Schiller et al. [17] and what is represented in Figure 4(c), we have

(12) c=23L,σ=13L.

In normal patients, *D*/*L* relation* (short axis/long axis)* in LV should vary from 0.45 to 0.62 [21]. Assuming this relation as a *δ* ∈ [0.45, 0.62] parameter, we have that *D*/*L* = *δ*, so

(13) $$\begin{array}{c} L=\frac{D}{\delta }. \end{array}$$

Thus it is possible to obtain the values of |*σ*| and *c* in function of *D*, if only we take (13) to (12), obtaining

(14) σ=D3δ,c=2D3δ.

With the values of *D*, we obtain the values of |*σ*| and *L*; consequently, the values of the TPS axle shafts, *a* and *c*, are obtained. From these measures and (8), we can estimate *A*_*σ*_(*S*) parameter, related to the LV surface area of normal patients.

### 2.3. Obtaining the Actual Medical Data

Macedo et al. [18] carried out a study in order to obtain the measures related to the right and left ventricles of Brazilian population (54 men and 53 women) by using MRI. In the LV, specially, they obtained the measures of the end-diastolic diameter (*D*_d_) and the end-diastolic volume normalized by the body area (*V*_dn_) in 107 normal individuals (asymptomatic and without cardiopathies).

For men and women, the data obtained in [18] to the total average and per age group are shown in Table 1, along with the calculated values of *a* by (10), which represents the measure of the smallest axis of TPS.

Through the data described in Table 1, considering three distinct cases for *δ* parameter and using (8), (13), and (14), it is possible to determine the approximations to the total average surface area of LV in women and men, besides doing this calculation by group age for both sex. The obtained results are presented and discussed in the next section.

## 3. Results and Discussions

During the simulations performed for the actual medical values of the short axis, once in healthy patients we have *δ* ∈ [0.45, 0.62], three values for *δ* were used: both ends of the interval and also 0.50, which is one of the relations most used in methods that calculate ventricular mass [8]. The average results obtained for |*σ*| and *A*_*σ*_(*S*) parameters are presented in Table 2, along with percentage differences per sex and their respective age group, for each one of the parameters.

The total average surface area obtained for men with *δ* = 0.45 consists of 145.40 cm^2^ which is only 3.71% shorter than the area obtained in [10], of 151 cm^2^. For men between 20 and 29 years, this difference is practically null, once the area of 151.39 cm^2^ was obtained. To the other age groups, the values are also relatively close to the one of [10]. Good approximations are also obtained when we consider *δ* = 0.50, specially to total average and to age groups until 49 years. For *δ* = 0.62, the value which is closer to Feng's result was for age groups of 30 to 39 years and for 40 to 49 years, with areas of 116.83 cm^2^ and the percentage difference of 22.63%.

We noticed, for women, a closer approximation in relation to the result of [10] to the situation where *δ* = 0.45 and to the age group of 30 to 39 years, in which the percentage difference is of 3.71%.

Yet, it is noticed that the values obtained for the LV surface area in healthy male patients are approximately 13.47% higher than the values obtained for female patients, in whatever the three cases of *δ* studied.

For the age groups separated by sex, this characteristic was kept, as well as to the three values of *δ* considered, however, with different values for each age group. We highlight that the major percentage difference observed is the fact that the male LV has an average surface area of approximately 34.5% greater than the female LV for age group of 40 to 49 years.

These constant percentage differences for the same age groups may indicate an important and interesting characteristic on the surface areas of the LV of men's and women's heart, allowing a greater geometric knowledge of the LV and can be used as one of the verification criteria of heart normality.

**Figure 5:**
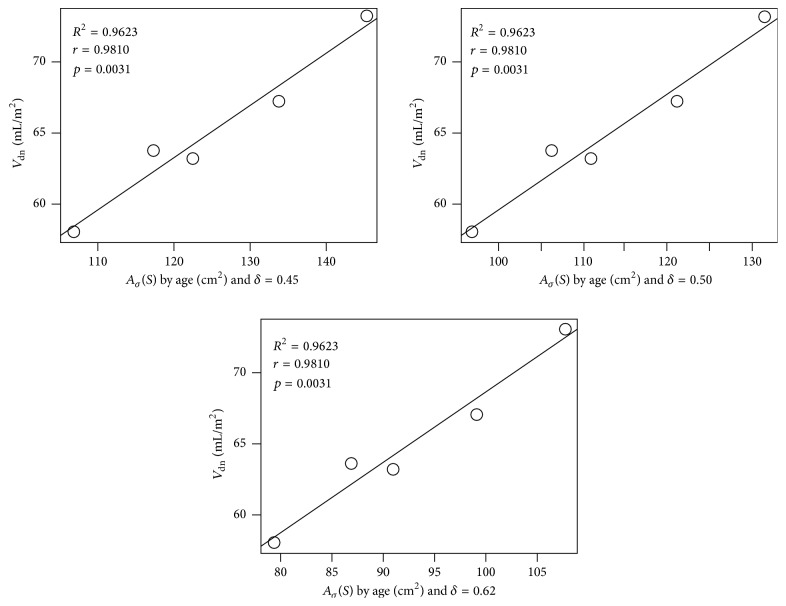
Dispersion and linear regression between the average surface area of LV and the end-diastolic volume normalized by the body area of women per age group.

We analyzed the correlations between the obtained surface areas and the end-diastolic volumes normalized by body areas. For the results of women age groups, we obtained *R*^2^ = 0.9623 and a positive strong correlation of *r* = 0.9810, for all of the three simulated values of *δ* (Figure 5). The obtained correlation is very close to the one obtained in [10], where *r* = 0.99. The significance of the determined correlation was validated by the *t*-test of Student for the level of significance *α* = 0.0031 and with 3 degrees of freedom.

**Figure 6:**
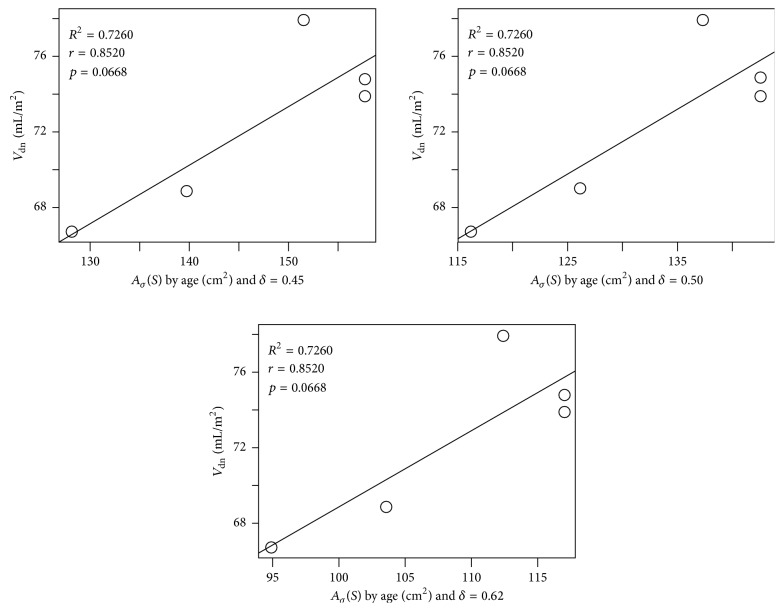
Dispersion and linear regression between the average surface area of LV and the end-diastolic volume normalized by the body area of men per age group.

For men we obtain *R*^2^ = 0.7260 and a strong correlation of *r* = 0.8520, with significance level *α* = 0.0668, also for all the three simulated values of *δ* (Figure 6).

*A*_*σ*_(*S*) parameter may also be applied in carrying out the Partial Left Ventriculectomy (PLV), which is a surgical procedure based on the resection of a slice of the LV free wall from patients with dilated cardiomyopathy in an advanced stage, so that the LV diameter can be reduced and the systolic function can be restored. This surgical procedure had its performance drastically reduced worldwide, but, on the other hand, it has been performed with strict criteria of selection, especially in Japan, and one of the problems in its realization is the lack of determination methods of the dimensions of the slice to be removed [22, 23]. Recently a mathematical model that allows this determination was developed and its practical application is basically dependent on the LV's surface area in normal patients [22, 24, 25].

Another possible application of this parameter is in the artificial organs development, where data relating to the surface area of cardiac chambers and heart, as a whole, can be considered while determining conditions to stop the growth of the extracellular matrix, so that the artificial heart developed has the specific characteristics of each patient [26–28].

## 4. Conclusions

In this work the demonstration of the formula to obtain the surface area of a TPS in Cartesian coordinates has been done, in order to associate its parameters with dimensions of axes of human LV. This type of modeling has an advantage over the modeling in ellipsoidal coordinates, once the dimensions needed to feed the model are linear and easy to obtain in more common exams, such as echocardiography.

Considering the resemblance of the LV and a TPS it was possible to obtain approximations of the LV's average surface in asymptomatic patients, verifying that, for the analyzed data, the percentage difference for surface areas of LV remained constant between men and women in age group of 20 up to above 60 years. These results are new and may be applied in many areas. In particular, they may be considered as new verification criteria of cardiac normality, be used to determine the dimensions of the cardiac slice to be removed during PLV, and also be used in developing artificial organs.

The work of Feng et al. [10] presents a single average result for the LV surface area, whose value is in accordance with the results obtained in our work. However, we present a greater range of possibility of results, once the verifications have been performed per age and sex, allowing analyzing these values for specific characteristics of the patients.

The strong correlations found between average areas and normalized diastolic volumes, for men and women, indicate that it may be possible to estimate one of these parameters, from the other one. In other words, a direct linear relation between them may exist.

However, a more detailed study is needed, with a higher number of patients, individually analyzed, aiming to verify if it is possible to better adjust the values of calculated areas and improve the correlations and the obtained significance levels. Thus, the obtained values and their characteristic of variations from a type of patient to another will be more accurate, allowing a greater success in its medical and biological applications.

## Figures and Tables

**Table 1:** Average of the reference measures for the LV of women and men and the calculated value *a*.

	Age group (years)	*D*_d_ (cm)	*V*_dn_ (mL/m^2^)	Values of *a* (cm)
Women	Total	4.6	68.5	2.30
	20–29	4.7	67.2	2.35
	30–39	4.9	73.2	2.45
	40–49	4.4	63.7	2.20
	50–59	4.2	58.0	2.10
	60–69	4.5	61.3	2.25

Man	Total	4.9	74.2	2.45
	20–29	5.0	78.1	2.50
	30–39	5.1	74.9	2.55
	40–49	5.1	73.9	2.55
	50–59	4.8	68.9	2.40
	60–69	4.6	66.6	2.30

Source: Macedo et al. [18] and the calculated value *a* by (10).

**Table 2:** Calculated values for |*σ*| and *A*_*σ*_(*S*) parameters, along with the percentage differences, per sex and age group.

*δ*	Age group (years)	Men		Women		Percentage differences (%)
		|*σ*| (cm)	*A*_*σ*_(*S*) (cm^2^)	|*σ*| (cm)	*A*_*σ*_(*S*) (cm^2^)	|(*A*_*σ*_(*S*)_men_ − *A*_*σ*_(*S*)_women_)/*A*_*σ*_(*S*)_women_|
0.45	Total	3.70	145.40	3.41	128.14	13.47
	20–29	3.37	151.39	3.48	133.77	13.17
	30–39	3.50	157.51	3.63	145.40	8.33
	40–49	3.49	157.51	3.26	117.24	34.35
	50–59	3.46	139.52	3.11	106.82	30.61
	≥60	3.54	128.14	3.33	122.63	4.49

0.50	Total	3.37	131.64	3.07	116.01	13.47
	20–29	3.03	137.06	3.13	121.11	13.17
	30–39	3.15	142.60	3.27	131.64	8.33
	40–49	3.14	142.60	2.93	106.14	34.35
	50–59	3.11	126.32	2.80	96.71	30.61
	≥60	3.19	116.01	3.00	111.02	4.49

0.62	Total	2.68	107.84	2.47	95.04	13.47
	20–29	2.45	112.29	2.53	99.22	13.17
	30–39	2.54	116.83	2.63	107.84	8.33
	40–49	2.53	116.83	2.37	86.96	34.35
	50–59	2.51	103.49	2.26	79.23	30.61
	≥60	2.57	95.04	2.42	90.96	4.49
